# Robust consensus nuclear and cell segmentation

**DOI:** 10.3389/fgene.2025.1547788

**Published:** 2025-07-15

**Authors:** Melis O. Irfan, Eduardo A. González-Solares, Tristan Whitmarsh, Alireza Molaeinezhad, Mohammad Al Sa’d, Claire M. Mulvey, Marta Páez Ribes, Atefeh Fatemi, Dario Bressan, Nicholas A. Walton

**Affiliations:** ^1^ Institute of Astronomy, University of Cambridge, Cambridge, United Kingdom; ^2^ Precision Breast Cancer Institute, Department of Oncology, University of Cambridge, Cambridge, United Kingdom; ^3^ CRUK Cambridge Institute, Li Ka Shing Centre, University of Cambridge, Cambridge, United Kingdom

**Keywords:** single-cell segmentation, imaging mass cytometry, multiplexed imaging, computer vision, bioinformatics

## Abstract

Cell segmentation is a crucial step in numerous biomedical imaging endeavors—so much so that the community is flooded with publicly available, state-of-the-art segmentation techniques ready for out-of-the-box use. Assessing the strengths and limitations of each method on a tissue sample set and then selecting the optimal method for each research objective and input image are time-consuming and exacting tasks that often monopolize the resources of biologists, biochemists, immunologists, and pathologists, despite not being the primary goal of their research projects. In this work, we present a segmentation software wrapper, coined CellSampler, which runs a selection of established segmentation methods and then combines their individual segmentation masks into a single optimized mask. This so-called “uber mask” selects the best of the established masks across local neighborhoods within the image, where both the neighborhood size and the statistical measure used to define what qualifies as “best” are user-defined.

## 1 Introduction

Segmentation is a prevalent challenge within medical imaging across a wide range of different modalities. Cell segmentation, in particular, enables counting, characterization, and spatial mapping of distinct cell types. A single analysis can require cell segmentation across tens to hundreds of tissue samples; the common practice is to select a single segmentation method based on its performance on a few samples and then assume that it will perform optimally across the entire cohort. However, in reality, different segmentation methods may work better for different tissue samples. Within a single sample, the best method may depend on the tissue region or cell type. Furthermore, even if time and staff resources would allow the option of manually annotating every tissue sample within a trial, Wiggins et al. (2024) have shown that variability between different expert annotators, or just the same annotator at different times, reduces the reproducibility of the subsequent analysis and scientific conclusions. In this work, we aim to capitalize on the strengths of multiple segmentation methods using a consensus technique.

Although traditional medical image segmentation techniques, such as thresholding or the watershed method, can be applied to sample data with a small number of user-defined input parameters, most modern methods typically rely on an initial set of human-annotated reference images. These images are used as either training samples for deep learning algorithms (Schmidt et al., 2018; Greenwald et al., 2022; Xiao et al., 2021; Stringer et al., 2021) or as Bayesian priors for maximum likelihood methods (Warfield et al., 2004). Artaechevarria et al. (2009) discussed the use of *a priori* human-annotated segmentation masks, referred to as “atlas” images, within expectation–maximization algorithms, and demonstrated that not only do multiple atlas images improve segmentation results for brain MRI data but so does altering the segmentation strategy across localized regions of the full image. Recent developments in image processing have placed researchers requiring nuclear segmentation in the fortuitous position of having numerous proficient algorithms available to them. The current challenge is how to appropriately select which technique to use for each region of interest. We introduce a consensus segmentation technique capable of considering the nuclear segmentation of any number of individual segmentation algorithms. This consensus technique determines which algorithm outperforms all others within user-defined subsections of the image and combines the optimum segmentations from different techniques into a single choice result for the image, which we refer to as the uber mask.

Consensus segmentation is not an unusual approach within computer vision for optimizing boundary detections. However, the majority of applications only require a per-pixel approach to voting as they are intended for use on continuous image data. Boiangiu and Ioanitescu (2013), for example, detailed a pixel co-association matrix approach, which successfully combines segmentation algorithms to produce clearly segmented representations of people, animals, scenery, etc. For per-pixel consensus voting, such as that proposed by Boiangiu and Ioanitescu (2013), there is the inherent risk that several pixels within a single feature will be identified as different from their neighbors. In the simple example of an apple sitting on a table in front of a wall, the segmentation might clearly show the apple, the table, and the wall as having clear boundaries, but within any one of those features, there may also be additional, segmented regions due to the range of pixel intensities across a single feature. Although this is not an unacceptable feature of continuous image processing, it would prevent further analysis within cell segmentation. If two cells were erroneously identified as occupying the same spatial area using a nuclear segmentation algorithm, then this would mislead any further interpretations of the tissue sample results.

For the specific case of single-cell nuclei detection, a consensus segmentation algorithm that maintains individual cell structures is required. Nguyen and Le (2022) moved beyond the standard per-pixel analysis and implemented a consensus vote that acknowledges the shape of the cell detected. As their technique takes into account the geometric and topological properties of the segmentation masks, it is referred to as “topological voting.” In this work, we extend their topological voting approach to be able to consider complex cell segmentation masks, which include multiple features, as opposed to the binary masks required for the blood vessel segmentation shown by Nguyen and Le (2022). In Section 2, we present an overview of our segmentation combination pipeline and introduce the publicly available segmentation algorithms to which we intend to apply our consensus vote. In this section, we also introduce various statistical criteria, which can be used to construct the optimum consensus mask; a strength of our pipeline is that it allows users to select different criteria for different image sets, based on their expertise and any cursory checks of the input images. Our pipeline is a general method applicable to any segmentation, and in Section 3, we present our results on a series of imaging mass cytometry and fluorescent imaging data. Section 4 presents the conclusion.

## 2 Materials and methods

### 2.1 Overview of the pipeline

**FIGURE 1:**
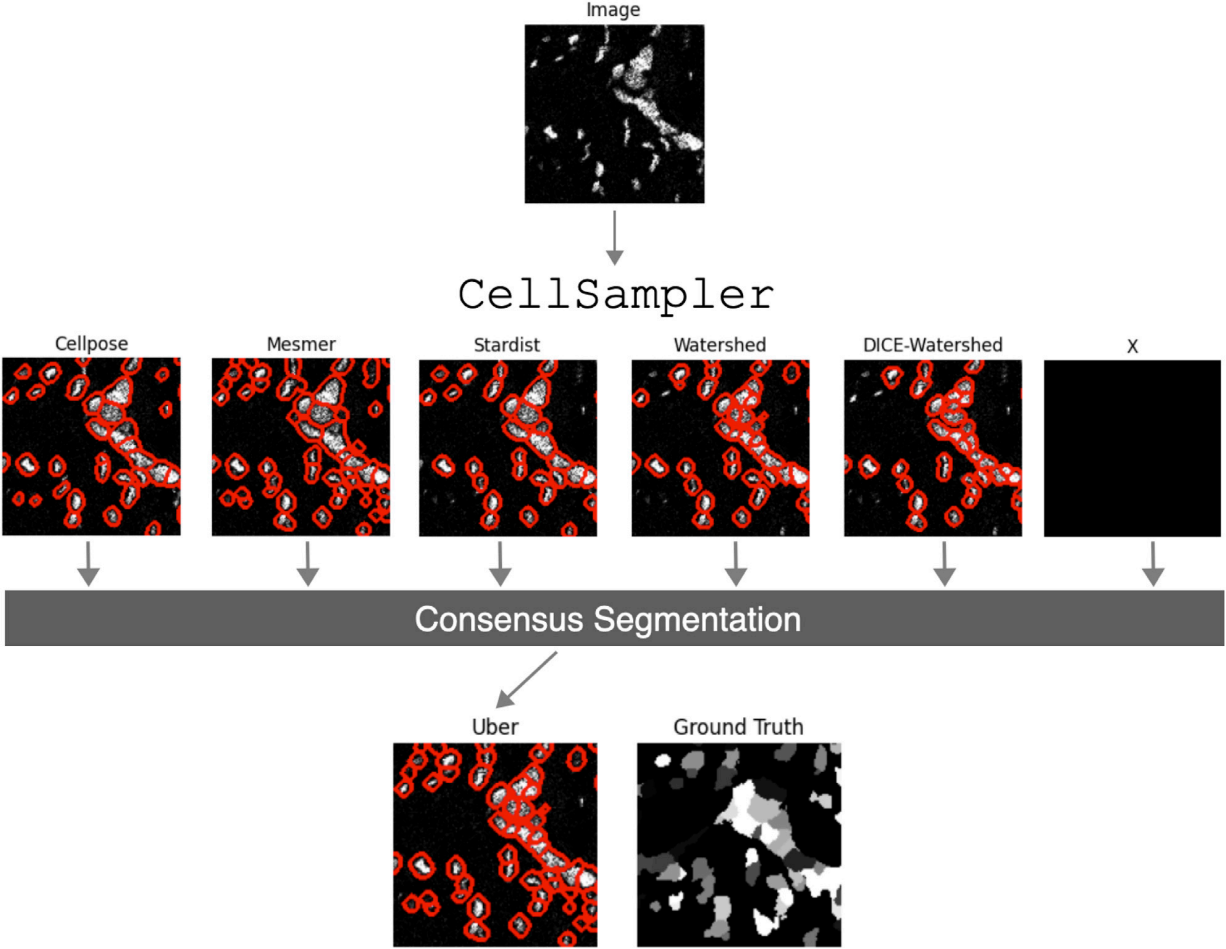
Schematic overview of the CellSampler pipeline. The input image is processed using various state-of-the-art segmentation algorithms, producing segmentation masks, which are then combined into a single optimal mask: the “uber mask.” The inclusion of an unknown method ‘X’ is to highlight the fact that CellSampler can, and will, be extended with additional segmentation methods.

Numerous segmentation algorithms are currently available for detecting cell boundaries within medical images, each with varying degrees of performance for different cell morphologies. The Python pipeline presented in this work, CellSampler, is a comparison and combination wrapper that aims to capitalize on recent advancements within nuclear segmentation by using a selection of state-of-the-art segmentation algorithms and combining their results into a single, optimized mask of cell nuclei. Figure 1 provides an overview of the CellSampler pipeline, which takes the form of a collection of Python scripts. CellSampler accepts input samples in Zarr format^1^ and produces segmentation masks in Zarr format and catalogs (lists) of individual cell properties in Parquet format^2^. IMC datasets can be converted to the Zarr format using the following code: https://gitlab.developers.cam.ac.uk/astronomy/camcead/imaxt/imc2zarr; alternatively, a Jupyter notebook is provided as part of the CellSampler code base, which details how to run the pipeline on an image in the form of a TIFF file. CellSampler runs a variety of nuclear segmentation methods, and more methods can easily be added to the pipeline framework. The code is extensible for the user to add their own segmentation methods or preprocessing steps; instructions for this functionality are provided within the code repository. The choice of how many and which methods to run, along with the user input parameters for each segmentation algorithm, is controlled by the user through a configuration YAML. The example data shown in Figure 1 are taken from the BRCA2 dataset, which is introduced in Section 3.1.

In this work, we explore the functionality of five established segmentation tools: Cellpose (Stringer et al., 2021), Mesmer (Greenwald et al., 2022), StarDist (Schmidt et al., 2018; Weigert et al., 2020; Weigert and Schmidt, 2022), a watershed segmentation, and an in-house implementation of Dice-XMBD (Xiao et al., 2021) followed by a watershed segmentation. As Cellpose, StarDist, and Mesmer have been optimized to perform on normalized input data, each input image is normalized between its first $\left(d_{\text{min}}\right)$ and 95th $\left(d_{\text{max}}\right)$ percentile values, as shown in Equation 1:

$$d_{\text{norm}}\left(p\right)=\left(d\left(p\right)-d_{\text{min}}\right)/\left(d_{\text{max}}-d_{\text{min}}\right),$$ (1)

and then clipped so that any values less than 0/greater than 1 are set to 0/1. Finally, local histogram equalization^3^ is applied to the scaled data to increase the image contrast. All the segmentation tools are run on identical images to set up a fair comparison environment; therefore, even if an algorithm has its own embedded normalization procedure, the image given to it has gone through the CellSampler preprocessing steps.

### 2.2 Cellpose

Cellpose is a deep learning algorithm that uses convolutional neural networks (CNNs) with the U-Net architecture to perform nuclear and cell segmentation. Cellpose has been trained on a variety of datasets and can also be retrained on specific images provided by the user. In this work, we use Cellpose3 and the built-in “nuclei” model type. This model has been pre-trained on several hundred microscopy and fluorescence images. As the option to load specific training weights exists within Cellpose3, it also exists with CellSampler. However, we choose to progress with the pre-trained models for this work. Cellpose also allows the user to input an expected cell diameter, and thus, we run two versions of Cellpose, one with an expected cell diameter of 6 pixels and another with an expected cell diameter of 10 pixels. Instead of requiring the selection of a single-algorithm input parameter, such as cell diameter, CellSampler allows users to evaluate several options simultaneously. We also alter the flow and probability thresholds to increase the number of nuclear detections until we are satisfied with the result; this is determined through visual inspection of a subset of five masks produced alongside the nuclear channel data. The ability to adjust cell diameter, flow, and probability thresholds is a functionality provided to the user through the CellSampler configuration YAML.

### 2.3 Mesmer

Mesmer is a segmentation algorithm that exploits the ResNet-50 CNN architecture and is trained on the TissueNet dataset (Barshir et al., 2012). In this work, we use Mesmer for nuclear segmentation (as opposed to whole-cell segmentation) and alter the image micron-per-pixel parameter to optimize our results. This scaling factor is a user input to the algorithm; Mesmer itself provides the scaling functionality. We use scaling factors of both 0.5 and 0.75 μm throughout, resulting in two implementations of Mesmer; these two factors were chosen in the same manner as the Cellpose parameter values.

### 2.4 StarDist

As with Cellpose, StarDist makes use of U-Net CNNs for nuclear and cell segmentation but, uniquely, enforces that the shape of each cell and nucleus can be characterized as a star-convex polygon. As the option to load specific training weights exists within StarDist, it also exists with CellSampler; however, we choose to progress with the pre-trained models for this work. The pre-trained model we select for use is the “2D_versatile_fluo” model, which is optimized for the detection of cell nuclei from 2D single-channel data.

### 2.5 Watershed

Our implementation of the watershed detection algorithm uses a Voronoi–Otsu binarization to separate the nuclei from their background, finds the local maxima within the image as a function of distance to the background, and finally uses the watershed functionality from scikit-image to determine individual nuclei from these maxima markers. For optimum performance, we first preprocess the image by identifying any pixels with a magnitude 10 times larger than the median values of their five nearest neighbors and reassigning those “hot” pixels to said median. We also subtract the background intensity from each circular region in the image with a radius of 75 pixels. The background intensity level is determined by smoothing the circular region pixels with a Gaussian beam.

### 2.6 Dice-watershed

As a possible improvement to the standard watershed detection, we also implement Dice-XMBD as a technique to segment the data before performing the watershed detection. This suggests that instead of performing watershed detection on the nuclear channel, it is more effective to apply it to the Dice-XMBD probability maps, which delineate each cell nucleus, cytoplasm, and the slide background. Dice-XMBD requires both a nuclear and a cytoplasm channel; however, as some imaging data either lack sufficient resolution or are not specifically stained for cytoplasm detection, CellSampler provides a “synthetic” cytoplasm channel.

The synthetic cytoplasm channel is formed by smoothing the nuclear channel image with a Gaussian and then using a Canny edge detector to find the edges meant to represent the cell detection. The nuclear and synthetic cytoplasm channels are combined and then smoothed again (to prevent any dips in intensity between the nucleus and cytoplasm edges) before being used as an input for Dice-XMBD. As we intended to implement the same U-NET architecture as Dice-XMBD and run it on the same trained model as, we ensured that our combined nuclear and cytoplasm data were preprocessed (including normalization and hot pixel removal) according to the Dice-XMBD methodology.

### 2.7 Consensus segmentation

Our consensus segmentation is an extension of the consensus approach proposed by Nguyen and Le (2022), tailored to fit the specific task of cell segmentation. The following is an overview of the algorithm proposed by Nguyen and Le (2022):

1. The local neighborhood of a single pixel is defined as the region within s pixels of the 2D pixel position ( x , y ), e.g., [ x−s : x+s , y−s : y+s ].
2. The Jaccard score is then calculated within the local neighborhood between all the contributing nuclear segmentation algorithms. The total Jaccard score for a single method is the sum of all the Jaccard scores between that method and the others.
3. The algorithm with the lowest total Jaccard score is eliminated from the process, and then, in a second round of voting, the new total Jaccard score is recalculated for the remaining contributing algorithms.
4. A value is assigned to the pixel, based on the algorithm, with the highest total Jaccard score.
5. Steps 2, 3, and 4 are repeated for every pixel within the 2D image.

We choose the minimum over maximum interpretation of the Jaccard score (J) between two segmentation masks ( $S_{m}$ and $S_{n}$ ) in our implementation, as shown in Equation 2:

$$J\left(S_{m},S_{n}\right)=\frac{\Sigma_{i,j}\;min\left(S_{m}\left(i,j\right),\;S_{n}\left(i,j\right)\right)}{\Sigma_{i,j}\;max\left(S_{m}\left(i,j\right),\;S_{n}\left(i,j\right)\right)}.$$ (2)

The type of segmentation performed by Nguyen and Le (2022) is, however, always binary (segmentation masks of only 0s and 1s), indicating that this is again a simplification of the problem the CellSampler consensus voting algorithm is trying to solve. In binary segmentation, the task is to determine tissue samples from a background of glass; in our case, we need to differentiate individual cells from each other and from the background. Additionally, we aim to allow the user to define the metric for an optimal segmentation based on a quick visual inspection of a few tissue samples. Chen and Murphy (2023) defined numerous metrics for evaluating the success of a potential segmentation in the absence of ground truth. Two of these metrics are 1) the number of cells detected and 2) the reciprocal of the natural logarithm of $\sigma \left(A\right)/\sqrt{n}$, where σ(A) is the standard deviation of cell areas and n is the number of cells. If the user had prior understanding of the number or consistency in size of the cells expected, then these two metrics would be valuable additions to the Jaccard score. It is worth noting, however, that using the incorrect metric can result in poor segmentation. For example, using the standard deviation of cell areas to inform the segmentation across a sample with a wide distribution of cell sizes would result in a segmentation biased toward a single common cell type. As the users have the greatest understanding of the tissue samples under analysis, we leave the choice of metric to them. Therefore, we propose the following alterations to the algorithm proposed by Nguyen and Le (2022):

1. The local neighborhood of a single pixel is defined as before, with s set to 40 pixels.
2. The “metric of merit” is then calculated within the local neighborhood between all the contributing nuclear segmentation algorithms. The choices of metric are
  a. the total Jaccard score (to compare the different nuclear masks, all nuclear detections are converted to binary masks),
  b. the number of unique nuclei, and
  c. $\frac{1}{\ln\left(\sigma \left(A\right)/\sqrt{n}\right)}$ .
3. For the algorithm with the highest metric of merit, we select the nuclear detections whose (x,y) pixel centers fall within the neighborhood and save those nuclei to an array that stores the nuclear pixel locations along with the method used to identify each nucleus.
4. Steps 2 and 3 are repeated for every neighborhood within the 2D image.
5. The array of all the saved nuclei from Step 3 is then checked for overlaps. Overlaps are first suggested as occurring when more than one nucleus center is closer to each other than the radius (assuming that the nucleus areas are the area of a circle) of the largest nuclei in the overlap group. All suggested overlaps are then checked against the original segmentation masks to determine whether an overlap truly occurs. If more than one method suggests a nucleus in the same location on the image [an overlap of more than 5 % of the $32^{\text{nd}}$ percentile (1 σ ) of segmented nuclei areas], then this is classified as an overlap.
  a. In the case of an overlap, another neighborhood is defined around the overlapping nuclei. The metric of merit is again calculated between all the methods, but this time the winner can only be chosen from the subset of methods that contributed a nucleus to the overlap group in the first place.
6. After the removal of overlaps, the remaining nuclear detections form the uber mask.

The functionality used to determine nuclear detection pixel centers and areas is taken from the skimage.measure library.

### 2.8 Data

We make use of the publicly available BRCA2 dataset (Jackson et al., 2020), which provides image mass cytometry (IMC) pathology images from breast cancer patients along with ground truth segmentations created using CellProfiler (Stirling et al., 2021). Part of the CellSampler pipeline involves using a CNN trained on 2D data with dimensions of 512 pixels by 512 pixels; therefore, to optimize its performance, input images are required to be of the same size. As the BRCA2 dataset is significant in size (746 images), we can afford to select larger images and then just crop them to the 512 pixel by 512 pixel area required for Dice-XMBD. We also expand our selection criteria to only include those images with over 400 cell detections, ensuring that the images processed contain a sizable number of cells to challenge the segmentation algorithms. These two requirements result in 258 images of 512 pixels by 512 pixels (pixel size 1 μm ), which are used to assess the nuclear segmentation algorithms.

Additionally, we include 49 fluorescent imaging samples from the Akoya Vectra 3.0 dataset, which was made publicly available by Aleynick et al. (2023). These data are derived from a selection of human tissue samples, including lung, breast, pancreas, colon, ovary, skin, tongue, and lymph node, and we selected the cropped images (400 pixels by 400 pixels) that were stained with the DAPI antibody and accompanied by ground-truth nuclear masks, which were annotated by specialists and approved by a pathologist. Finally, we show segmentations for two images provided by the IMC facility within the CRUK Cambridge Institute. Both images are obtained from mouse tissue, with one being lung tissue and the other being ovarian tissue.

## 3 Evaluation

Within this analysis, we use four scores to assess the success of each nuclear segmentation mask: the recall, precision, F1, and Jaccard scores. Each score ranges from 0 to 1, with 1 being the best possible result. These scores all rely on a ground-truth segmentation mask, which is used to assess possible detections as true positives (TPs), fake positives (FPs), and fake negatives (FNs). The recall, precision, F1, and Jaccard scores can be calculated using Equations 3–6, respectively:

$$Recall=\frac{TP}{TP+FN},$$ (3)

$$Precision=\frac{TP}{TP+FP},$$ (4)

$$F1=\frac{2\times Precision\times Recall}{Precision+Recall},$$ (5)

$$Jaccard=\frac{TP}{TP+FP+FN}.$$ (6)

We rely on the intersection over union (IOU) measurement between a ground-truth nucleus and a predicted nucleus to decide how to classify each of the predicted nuclei as TP, FN, or FP. For the example of a ground-truth mask containing $N_{o}$ nuclei and a mask predicted using a nuclear segmentation algorithm containing $N_{p}$ nuclei, an IOU matrix of the size of $N_{o}$ by $N_{p}$ would be constructed. For computational ease, we do not calculate every element of the IOU matrix; instead, we assume that ground-truth and predicted nuclei with centers (x,y) more than 80 pixels apart will not intersect and therefore assign them an IOU value of 0.

First, we assessed the predicted nuclei: all those with IOU values <0.1 were designated FPs. A predicted nucleus with IOU values >0.1 was marked as a TP match for the one ground-truth nucleus with which it shared its highest IOU value. That ground-truth nucleus would then be marked as matched. If the highest IOU (to two decimal places) for a predicted nucleus matched more than one ground-truth nucleus, then this predicted nucleus was marked as a merge error. Similarly, if the highest IOU value for a predicted nucleus was matched to a ground-truth nucleus that was already marked as matched, then this would be a split error. Merge (one predicted nucleus matched to many ground-truth nuclei) and split (multiple predicted nuclei matched to one ground-truth nucleus) errors were both counted as FPs. Finally, we went through the ground-truth nuclei, and any of these that did not have an IOU value >0.1 with a predicted nuclei were marked as FNs.

### 3.1 BRCA2

**FIGURE 2:**
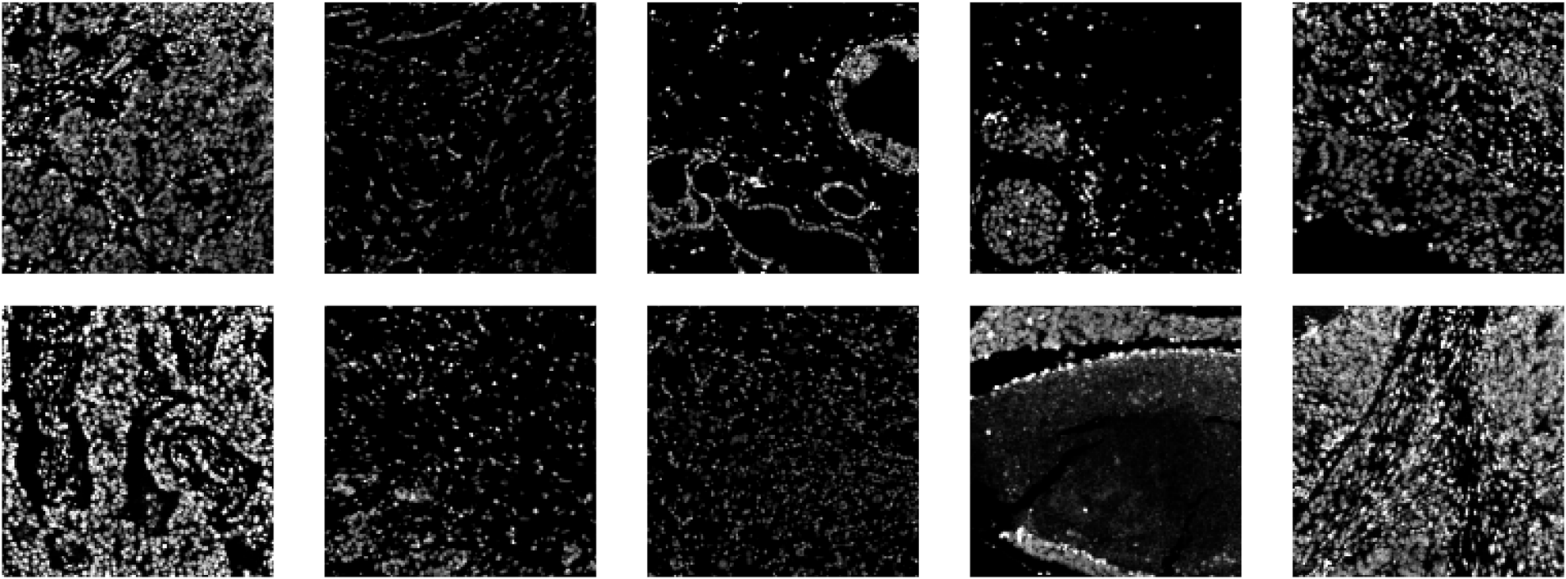
Ten example images (nuclear channel) from the BRCA2 dataset.

To represent the nuclear channel, we use the average between two DNA marker channels: those stained with iridium 193 and 191. Figure 2 shows a subset of the nuclear channel IMC images from the BRCA2 dataset; a variety of morphologies and intensities can be observed, even within this small subset. As the ground-truth segmentations are provided for the whole cell (not just the nucleus), we expanded our nuclear prediction masks each by a fixed number of pixels across all the images. As it can, sometimes, be challenging to find a clear cytoplasmic marker present within the IMC staining panels—and in some cases none are available—we chose to implement the option of expanding the nuclear detections to represent cell detections.

**FIGURE 3:**
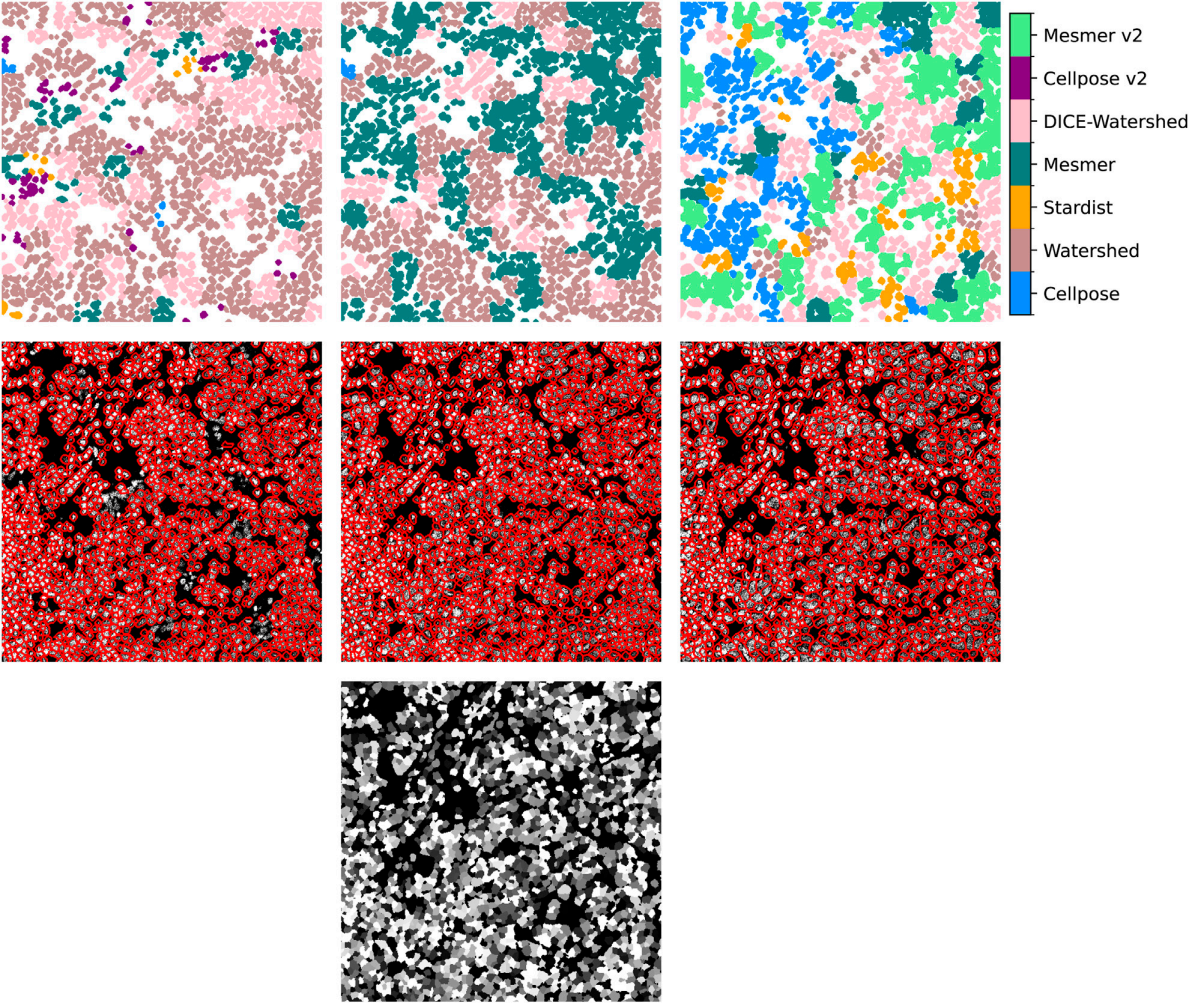
Top row: the cell detections selected from each method using the uber mask for our example image. Middle row: the uber mask contours in red, overlaid on the input image. From left to right: the uber mask made using 1) the reciprocal of the natural logarithm of the standard deviations of the nucleus areas, 2) the number of nuclei detected, and 3) the Jaccard score as the score of merit. Bottom row: the ground-truth mask.

In this work, Cellpose and Cellpose v2 refer to Cellpose used with a user-specified cell radius of 10 pixels and 6 pixels, respectively. Mesmer and Mesmer v2 refer to Mesmer used with user-specified scaling factors of 0.75 and 0.5 μm , respectively. The top row of Figure 3 shows the cell detections within the uber mask for an example image. Each segmented cell is colored according to the prediction algorithm that provided the segmentation. The three plots, from left to right, are the uber mask results for the three possible metrics of merit: 1) the reciprocal of the natural logarithm of the standard deviations of the nucleus areas, 2) the number of nuclei detected, and 3) the Jaccard score. The middle row shows the uber mask contours in red, overlaid onto the input image. It is clear that the choice of metric makes a significant difference to the uber mask produced. Although standard processes usually involve making an educated guess on which segmentation method to choose, the CellSampler pipeline allows the user to make an informed decision on what segmentation quality they would like to propagate into their data analysis from all the available methods. For the example shown in Figure 3, the user can visually verify that the uber mask produced using the criterion of the number of nuclei detected best represents the ground truth. By examining the choice of masks for a couple of images and making a couple of ground-truth annotations, it would be observed that under-segmentation is the key problem across the majority of regions and methods used in this analysis.

**FIGURE 4:**
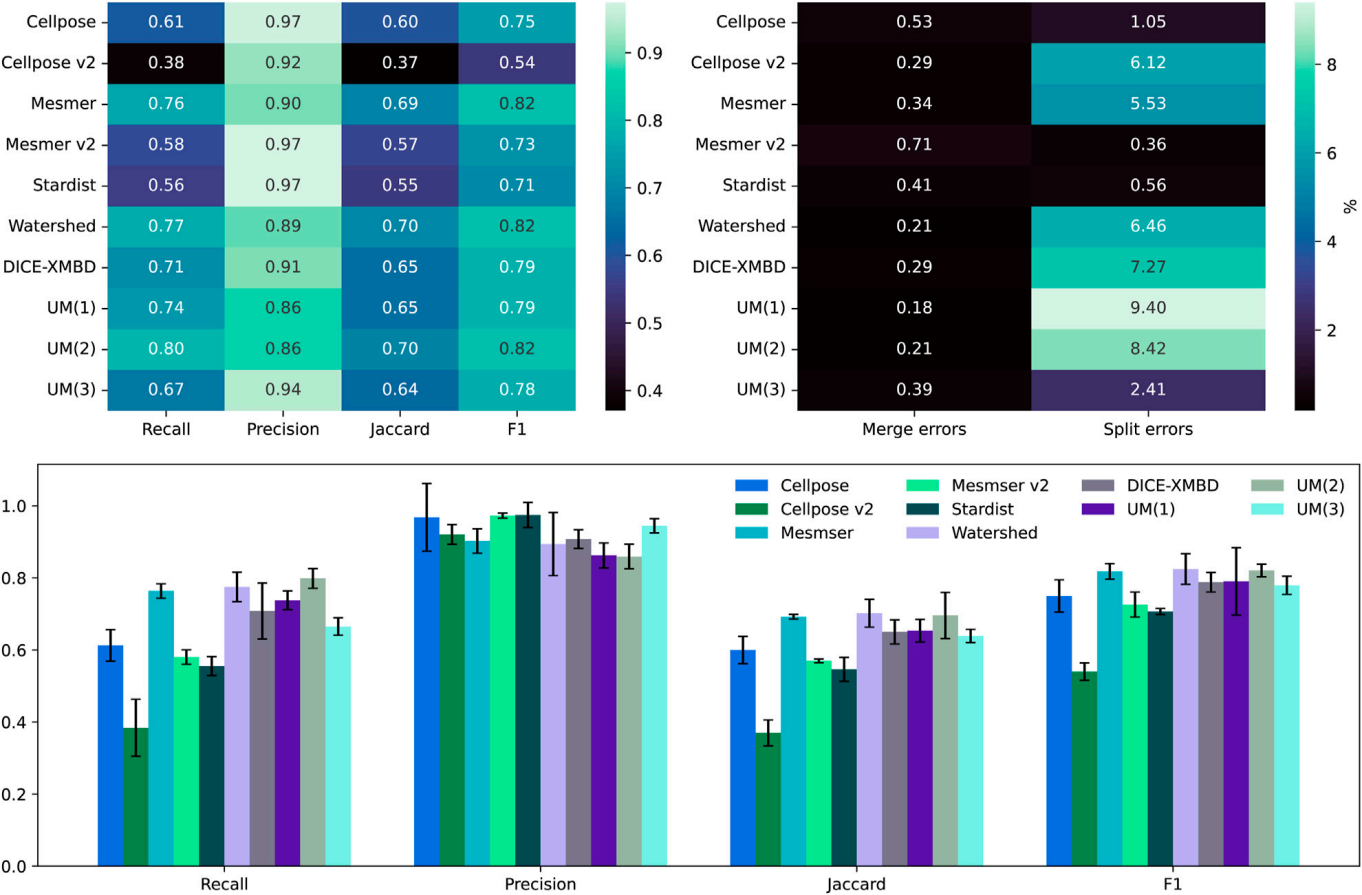
Top left: comparison between the median scores for all the nuclear masks of the IMC dataset. Top right: the percentage of split and merge errors in our 258 images. Bottom row: the same as the top left but as a bar chart, where the error bars are the mean absolute deviation values for the scores.

As shown in Figure 4, the recall, precision, Jaccard, and F1 scores are calculated for all five segmentation algorithms, along with the three versions of the uber mask:

• UM(1): the mask formed using the reciprocal of the natural logarithm of the standard deviations of the nucleus areas as the criteria of merit,

• UM(2): the mask formed using the number of nuclei detected as the criterion of merit, and

• UM(3): the mask formed using the Jaccard score as the criterion of merit.

The recall score is focused on how many true positives can be detected using an algorithm, and in this case, the methods that produce the largest number of overall cell detections tend to perform the best. Cellpose v2 and StarDist, as used in their “vanilla” mode, i.e., without any specific training on this dataset, perform the worst, having identified the fewest number of cells altogether. Under-segmentation is perhaps one of the easiest issues to identify without ground-truth masks, through visual inspection of segmentation contours overlaid on the nuclear image. The uber mask, version UM(2), which maximizes the number of nuclei detections, has, unsurprisingly, the highest recall score. The precision score, however, focuses on how accurate an algorithm’s cell detections are and actively penalize methods that propose false positives. Conversely, the two methods that produce the fewest cell detections have the highest precision as generating fewer total predictions also results in fewer false-positive suggestions. The Jaccard and F1 scores are more comprehensive as they both include an assessment of true positives, false positives, and false negatives. For both these scores, the watershed, Mesmer, and UM(2) uber masks have the highest scores, only differing from each other by 0.01, which is well within their error bars (shown in the lower plot of Figure 4) across the full dataset of 258 images. For all the methods, it is observed that in the BRCA2 images, split errors (multiple predicted nuclei matching a single ground-truth nucleus) are far more common than merge errors (a single predicted nucleus matching multiple ground-truth nuclei).

The results presented in Figure 4 relate to an IOU threshold of 0.1. Table 1 shows that changing this threshold value alters the results but not the performance of the uber mask. As the threshold defines the overlap percentage required for a match between the segmentation and the ground truth, increasing the threshold results in fewer matches and fewer split and merge errors. The top two methods in this analysis remain the same—Mesmer and watershed—and the UM(2) uber mask performs as well as these methods within their margin of error.

**TABLE 1:** F1 and Jaccard scores for Mesmer, watershed, and the UM(2) uber mask using different IOU threshold values and different pixels sizes to define the local neighborhood.

IOU	F1			Jaccard		
	Watershed	Mesmer	UM(2)	Watershed	Mesmer	UM(2)
0.1	0.82	0.82	s = 20 ↦ 0.81	0.70	0.69	s = 20 ↦ 0.69
			s = 40 ↦ 0.82			s = 40 ↦ 0.70
			s = 80 ↦ 0.82			s = 80 ↦ 0.70
0.3	0.81	0.80	0.81	0.69	0.67	0.67
0.5	0.67	0.67	0.65	0.50	0.50	0.48

It is worth noting that the metric of merit is not the only user-defined parameter required for the creation of the uber mask; the user must also choose the size of the local neighborhoods within which the metric of merit is calculated. For the results shown in this section, a pixel size of 40 was used to define the local neighborhoods. We also investigated the use of a neighborhood size of 20 and 80 pixels for the UM(2) uber mask; the scores for each pixel size (s) are stated in Table 1. The scores are all consistent to within 0.01, regardless of the neighborhood size used, signifying that the uber mask is fairly robust to the choice of local neighborhood—as long as the choice is larger than the average expected cell radius.

Splitting the image into neighborhoods smaller than a single cell would prevent the CellSampler pipeline from converging on a solution for a single-cell prediction. For the image analysis carried out in this paper, typical cell diameters were approximately 10 pixels, and to ensure that at least two cells were present within each neighborhood, we restricted the minimum neighborhood size to 20 by 20 pixels. Conversely, setting the neighborhood size too large would result in a single method being chosen across a large number of pixels, thus defeating the purpose of CellSampler, which aims to benefit from combining the strengths of multiple methods. We chose to investigate a maximum neighborhood size of 80 pixels as our samples were only slightly more than six times larger in length (512 pixels). The user has control over the neighborhood size, but we have shown that this parameter has only a minor impact on the segmentation when used within a logical range.

The Wilcoxon signed-rank test can be used on the concatenated Jaccard and F1 scores for each method to identify statistically significant differences between the three methods that possess the highest average F1 and Jaccard scores. The Wilcoxon *p*-values between both the watershed method and UM(2) and the watershed method and Mesmer are negligibly small. The Wilcoxon *p*-value between UM(2) and Mesmer, however, shows a notable difference at 0.054. The negligible *p*-value between the watershed and Mesmer is interesting as these are very different segmentation algorithms by design. However, it appears that they concur on which images are straightforward to segment and which are more troublesome. The uber mask will be constructed primarily from the ‘best’ methods, so it is unsurprising that there are strong correlations within the individual image Jaccard and F1 scores between UM(2) and both the watershed and Mesmer. The negligible *p*-value between the watershed method scores and the UM(2) scores reveals that the uber mask for the BRCA2 dataset consists mainly of watershed segmentations.

### 3.2 Fluorescent imaging data

We apply CellSampler to the DAPI channel of the multiplex fluorescent imaging samples to perform nuclear segmentation. Since the images are 400 pixels by 400 pixels, we choose to apply all the methods except Dice-XMBD as this method works optimally on images of 512 pixels by 512 pixels.

**FIGURE 5:**
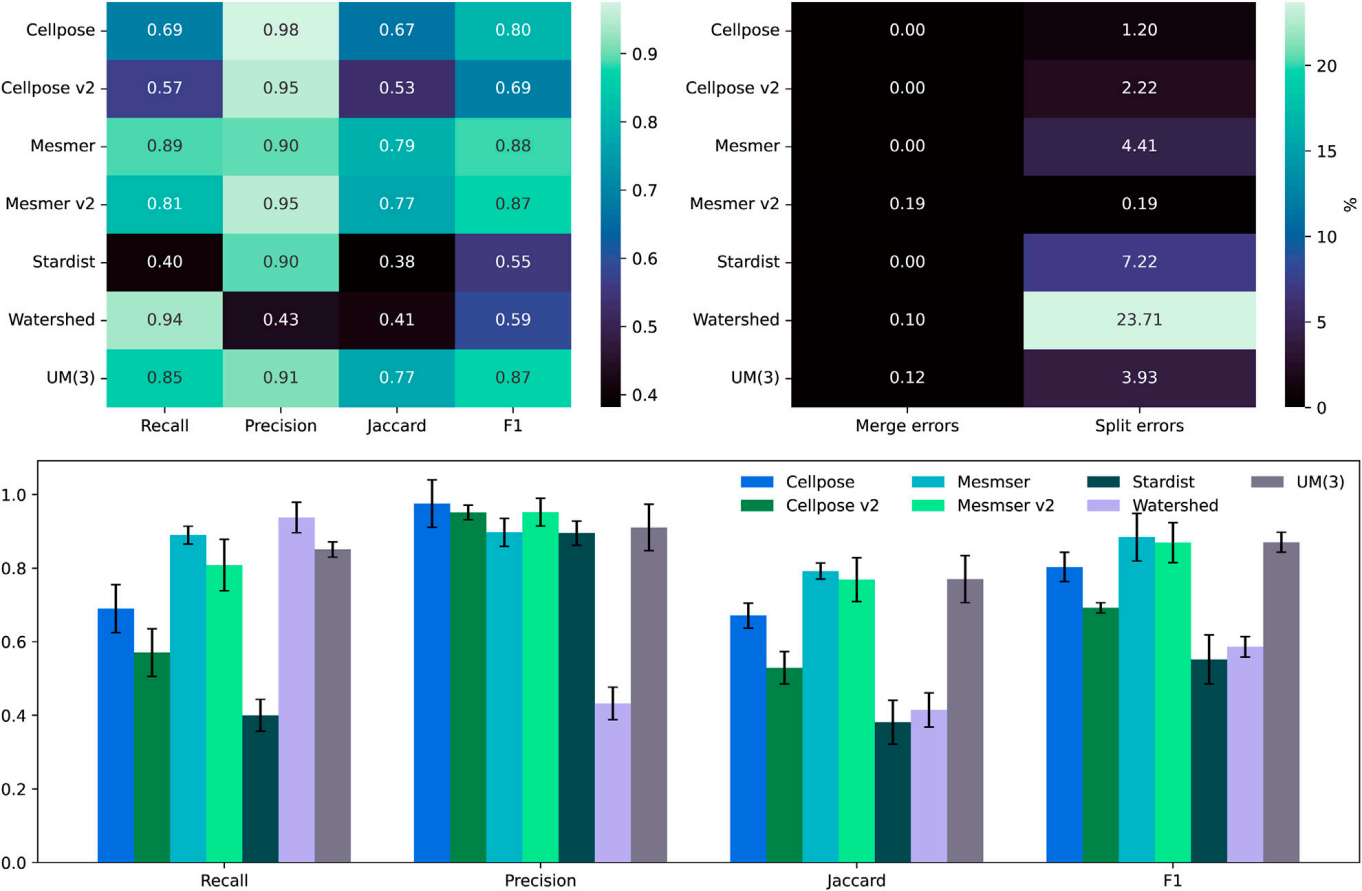
Top left: comparison between the median scores for all the nuclear masks created for the fluorescent imaging dataset of 49 images. Top right: the percentage of split and merge errors in our 49 images. Bottom row: the same as the top left but as a bar chart, where the error bars are the mean absolute deviation values for the scores.

Figure 5 shows the recall, precision, F1, and Jaccard scores between the annotated nuclear masks and our segmentation methods. As before, we focus on the Jaccard and F1 scores as they assess the numbers of true positives, false positives, and false negatives. We use an IOU threshold of 0.1, and for the bar chart, we plot the median score using the median absolute deviation for the error bars. For this dataset, the highest Jaccard and F1 scores are attained using the Mesmer algorithm (both with a 0.5 and 0.75 μm image resolution) and the UM(3) uber mask. These three methods display the same Jaccard and F1 scores within their error bars, showing that the uber mask functionality performs equally well on both IMC and fluorescent imaging data.

### 3.3 Qualitative results

**FIGURE 6:**
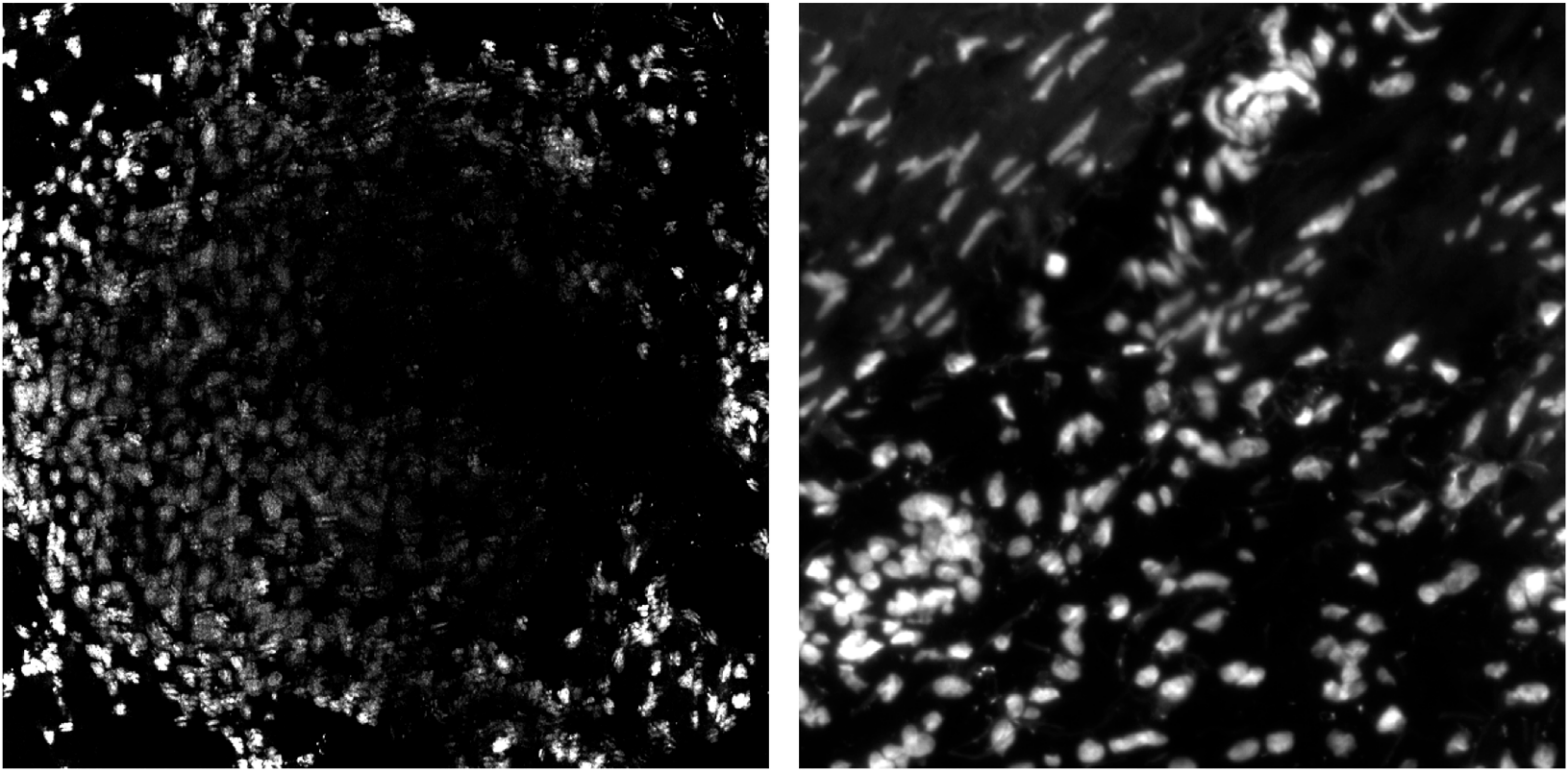
Two IMC images of mouse tissue: lung tissue on the left and ovarian tissue on the right.

In this section, we take a closer look at the performance of the uber mask on two specific IMC images: one showing a section of the lung and another showing a section within the ovaries. We perform nuclear segmentation on the average between two nuclear maker channels: one stained with iridium 193 and another with iridium 191. We have no ground-truth annotations for these two IMC images; instead, we assess the nuclear detections visually using segmentations overlaid onto the input images. Figure 6 shows both the lung and ovarian IMC images. The lung sample was chosen for this analysis because it contained a metastatic lesion that presented fainter, more difficult-to-segment nuclei. The ovarian sample presents both thin, long nuclei and the standard circular nuclei observed in the BRCA2 samples.

**FIGURE 7:**
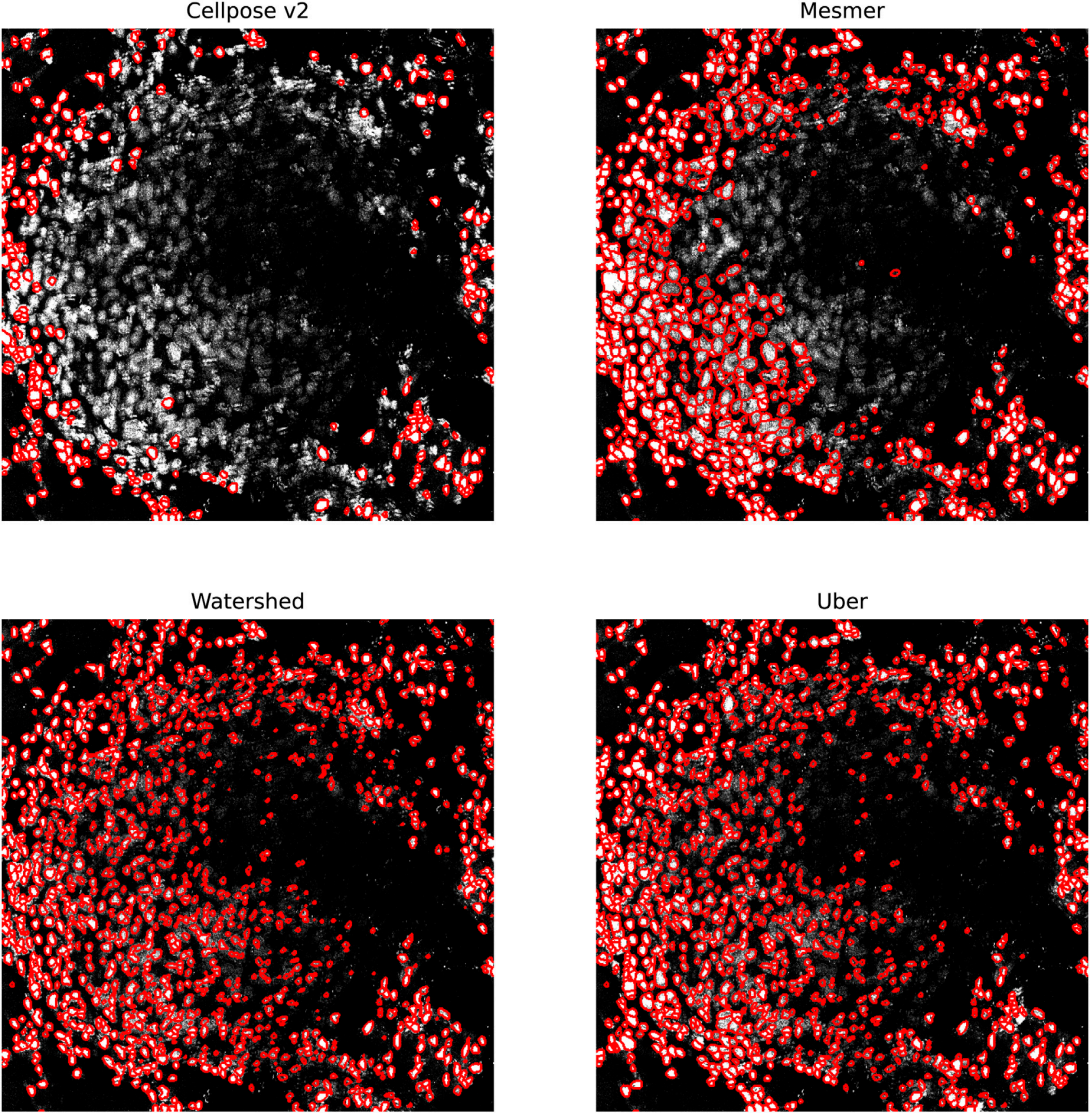
Specific region of the lung IMC sample. The nuclei segmented through Cellpose v2, Mesmer, watershed, and the uber mask are shown as red contours overlaid onto the nuclear channel data.

**FIGURE 8:**
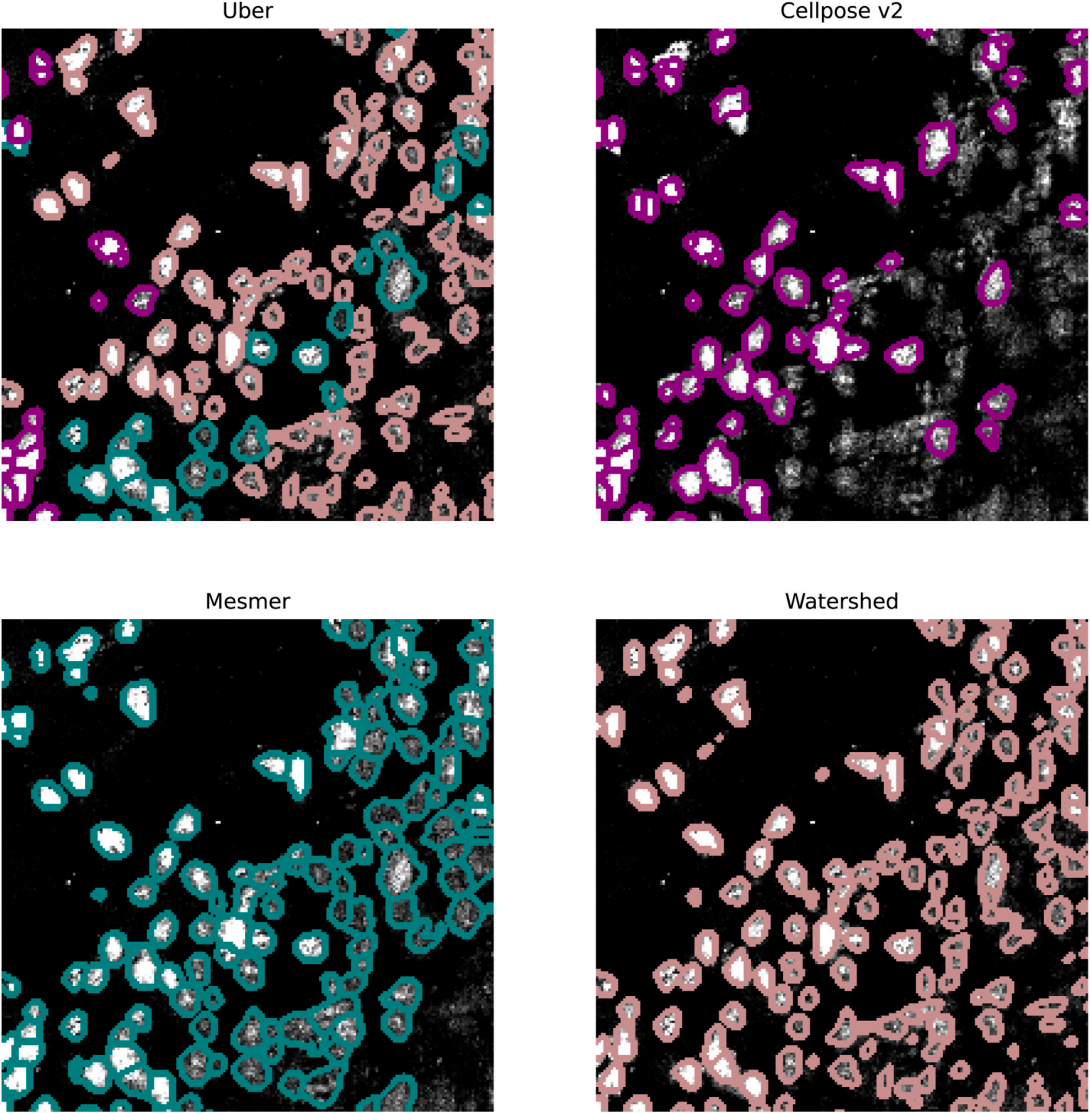
Specific region of the lung IMC sample. The top left image shows the uber mask nuclear detections as contours over the original image. The contours are colored according to the method that detected them. The top right, bottom left, and bottom right images show the Cellpose v2, Mesmer, and watershed detections in purple, teal, and light brown, respectively.

In Figure 7, we focus on the metastatic lesion within the lung. For this image, under-segmentation was a problem, and therefore, the uber mask was formed using the number of nuclear detections for the criteria of merit (neighborhood size of 40 pixels). The three algorithms that contributed to the majority of the uber mask detections were Cellpose v2, Mesmer, and watershed; the nuclear detections from these three methods and for the uber mask have been plotted as red contours over the input image. In Figure 8, we illustrate exactly how the uber mask is constructed by showing several nuclear detections within the lung sample. Within the region shown in Figure 8, the uber mask consists of nuclear segmentations from Cellpose v2, Mesmer, and watershed. The segmentations for Cellpose v2 are shown as purple contours overlaid onto the input image, the Mesmer segmentations are shown as teal contours, and the watershed segmentations are shown in light brown. The uber mask is made up of contours from all of these methods, choosing Mesmer and Cellpose v2 over the watershed technique for the specific areas where they detect a higher number of nuclei than the watershed technique.

**FIGURE 9:**
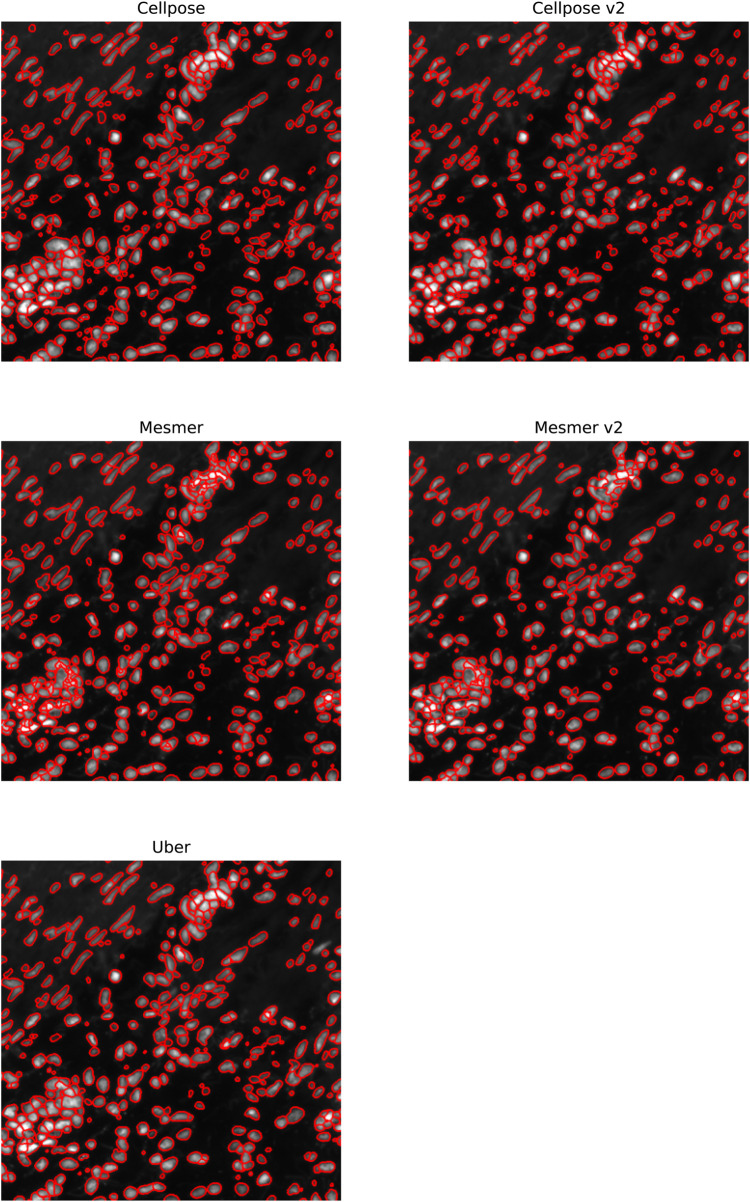
Specific region of the ovarian IMC sample. The nuclei segmented through Cellpose, Cellpose v2, Mesmer, Mesmer v2, and the uber mask are shown as red contours overlaid onto the nuclear channel data.

**FIGURE 10:**
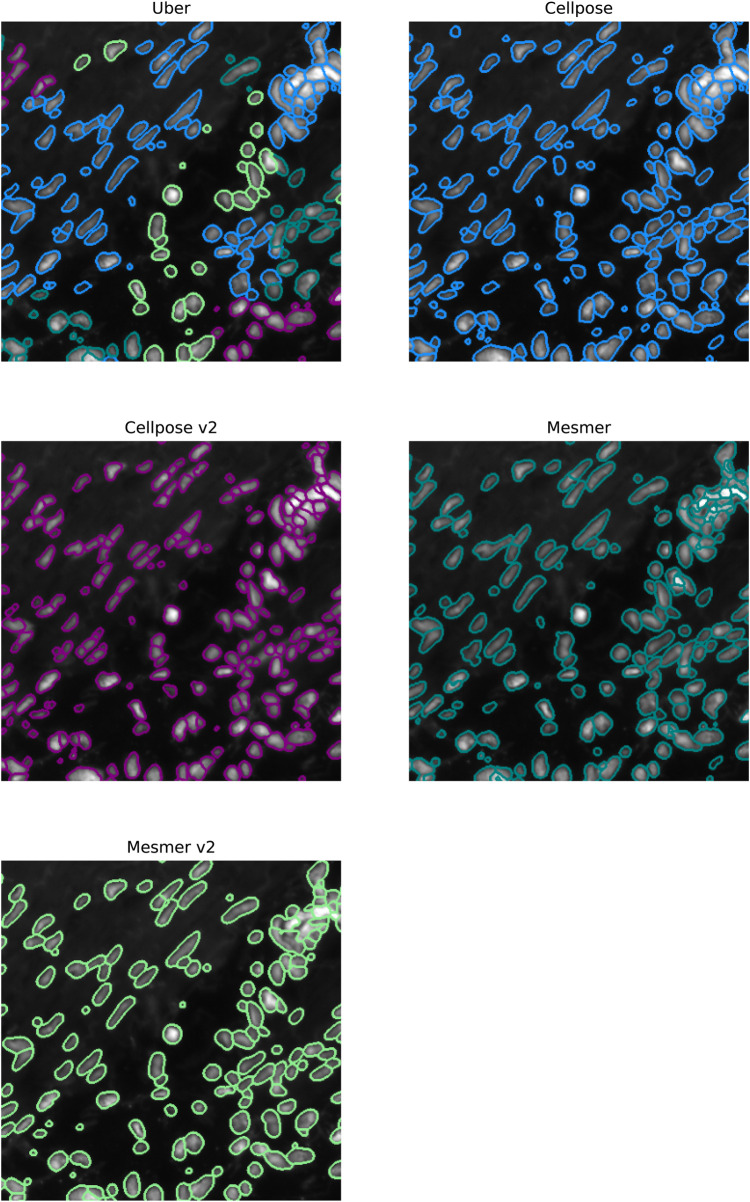
Specific region of the ovarian IMC sample. The top left image shows the uber mask nuclei detections as contours over the original image. The contours are colored according to the method that detected them. The top right, middle left, middle right, and bottom left images show the Cellpose, Cellpose v2, Mesmer, and Mesmer v2 detections in light blue, purple, teal, and light green, respectively.

In Figure 9, we focus on a fairly typical region within the ovarian sample; this tissue sample was chosen as it contains both circular and elliptical-shaped nuclei. For this sample, under-segmentation was not observed to be a problem; the challenge for the segmentation algorithms was in simultaneously identifying two strikingly different cell structures. The uber mask was formed using the Jaccard score for the criteria of merit (neighborhood size of 40 pixels). The four algorithms that contributed to the majority of the uber mask detections were Cellpose, Cellpose v2, Mesmer, and Mesmer v2; the nuclear detections from these four methods and for the uber mask have been plotted as red contours over the input image. For the specific region shown in Figure 10, the uber mask consists of nuclear segmentations from Cellpose, shown as light blue contours overlaid onto the input image, Cellpose v2 given as purple contours, Mesmer segmentations shown as teal contours, and Mesmer v2 segmentations shown in light green. The uber mask can strike a balance between identifying all the nuclei clearly visible to the eye while avoiding over-segmentation and the misclassification of background noise as a genuine signal.

**FIGURE 11:**
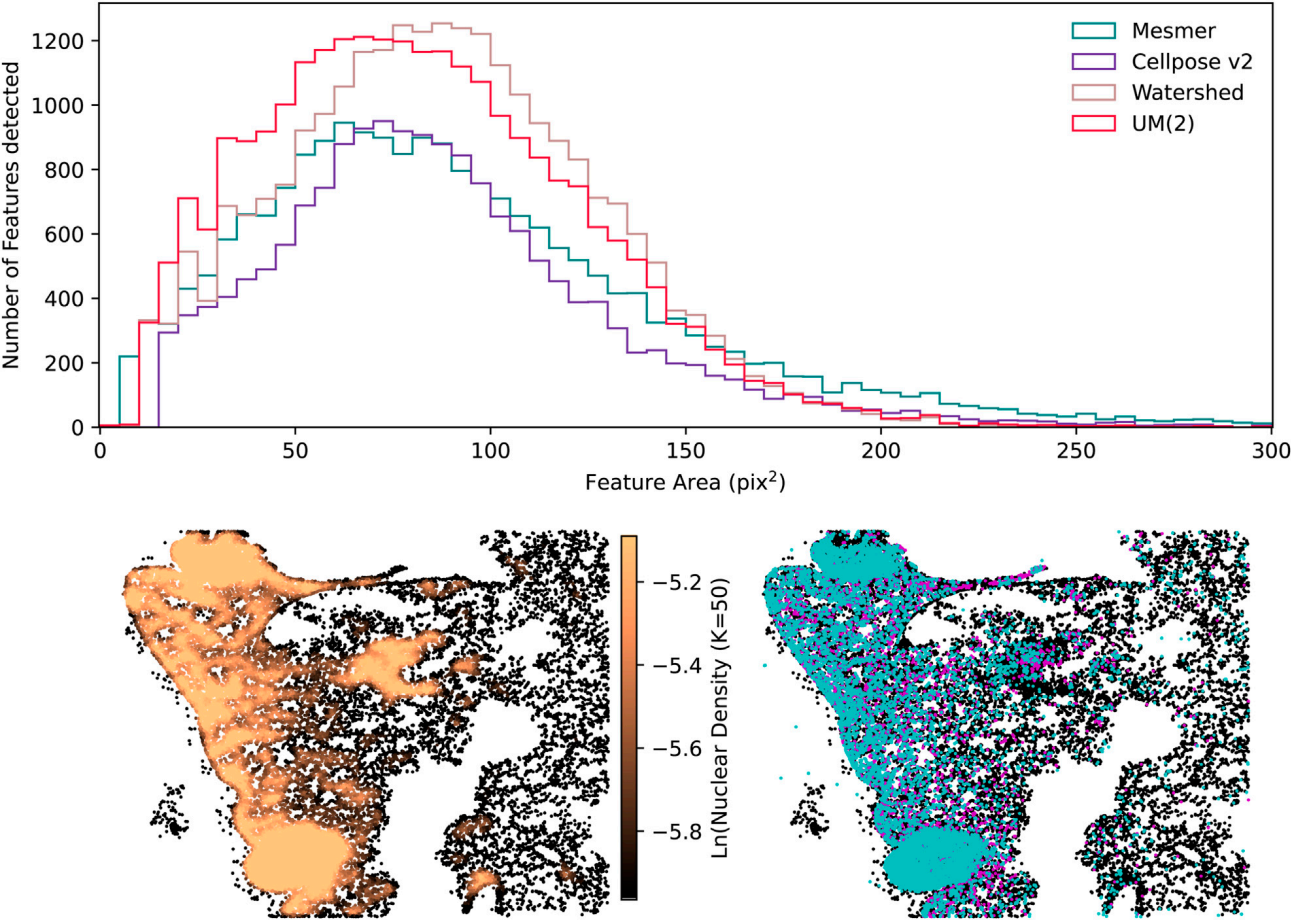
Examples of some diagnostic plots produced for the segmentation of the IMC mouse-lung tissue. The top row shows the histogram distribution of nuclear areas. On the bottom row, the left-hand plot shows the 50 nearest-neighbor nuclear density plot, and the right-hand plot shows the brightest nuclei in the CD68 channel in cyan and the CD11b channel in magenta, overlaid onto all the detected nuclei (shown in black). The figures in the bottom row were generated using the UM(2) segmentations.

These two examples explicitly show the advantage of a consensus segmentation method in optimally capturing as many genuine nuclear detections as possible while avoiding over-segmentation for a variety of cell types. Assessing segmentations without expert human annotations to serve as ground-truth masks can sometimes be too complex to perform by eye alone; therefore, the CellSampler code repository includes diagnostic plots to help researchers assess performance. Figure 11 shows a sample of diagnostic plots for the IMC mouse-lung image, the histogram distribution for nuclei areas, a k-nearest neighbor nuclear density plot, and the mean intensities per nucleus within the two IMC channels. The UM(2) uber mask and the watershed technique can detect the highest number of nuclei, but while the Cellpose v2, Mesmer, and UM(2) nuclei share similar areas, the watershed method finds more nuclei with slightly larger areas. Examining cell counts and cell areas provides insights into which method identifies the highest number of nuclei without segmenting individual nuclei into several smaller pieces. The uber mask nuclear density plot shows $N/\left(\pi R^{2}\right)$ , where N is the number of nearest neighbors (50 in this case) and R is the average distance between these neighbors; this plot can be used to assess the regions within the tissue sample that contain the highest number of nuclei across the smallest distances. Additionally, any multiplex dataset will contain several channels of information, so the intensities measured across different channels can be calculated using the segmentation determined from the nuclear channel. An example of this is shown in the bottom left plot of Figure 11, where the brightest nuclei from channels CD68 (a macrophage marker) and CD11b (a monocytic marker) are plotted in cyan and magenta, respectively. Here, the term “brightest” means the nuclei that have a mean intensity value larger than the $68^{th}$ percentile intensity for the whole tissue sample in that channel.

### 3.4 Technical details

CellSampler works on sample directories that can contain multiple regions of interest (ROIs). For a sample directory that contains four ROIs of sizes (3,054 × 3551), (1,529 × 3561), (4,251 × 2161), and (2,793 × 3926) pixels, the runtime using 40 CPUs, 1 GPU, and 55 GB of RAM is 1 h and 40 min. This operational time includes the production of segmentation masks for five different methods ( $∼10^{4}$ single nuclei detections), the formation of the uber mask, and the documentation of notable nuclei properties (area, intensity, and orientation, for example) within 49 IMC channels. Running a single method only, such as Cellpose, would produce four segmentation masks (for the four ROIs) within 12 min. The approach of running multiple methods and using statistical properties to highlight the best methodologies across different sample regions was taken to save staff hours at an earlier stage of segmentation method selection.

CellSampler can be installed using the Python package management system pip; upon installation, CellSampler will install all the sub-packages required for a successful run. For this initial software release, these include the segmentation tools Cellpose, Mesmer, StarDist, Watershed, and DICE-XMBD.

## 4 Discussion

We have presented a new consensus voting technique that is capable of combing multiple segmentation algorithms for the segmentation of a single image. Our consensus voting is applicable to a wide range of spatial genomic imagery; we show results for IMC and fluorescence images in this study. Algorithms such as Cellpose and StarDist have been trained on H & E images, so CellSampler can also be used for these types of images. The consensus technique presented in this work specifically divides the single image into local neighborhoods, allowing for different algorithms to lead the segmentation across different, localized regions. We have shown that, for the BRCA2 dataset, the consensus uber mask, which votes based on the number of cell detections within a local neighborhood, produces the joint highest F1 and Jaccard scores, along with the winning algorithms (Mesmer and watershed for this analysis). For the Aleynick et al. (2023) dataset, the consensus uber mask, which votes based on the Jaccard score within a local neighborhood, produces the joint highest F1 and Jaccard scores, along with the winning algorithm (Mesmer for this analysis). We have also demonstrated the ability of the uber mask to capture the optimal number of nuclear detections across a selection of different IMC images presenting different cell types and supply diagnostic plots, such as cell area and channel intensity distributions, to help assess the segmentation masks when no ground truths are available.

The CellSampler pipeline can optimize cell segmentation for large sample size image sets by enabling researchers to quickly test a variety of segmentation algorithms, identify the key strength in what would appear to be the optimum method through a visual comparison of several segmented images, and then construct a reliable segmentation for all of their hundreds/thousands of images through an uber mask optimized to make choices based on the user-selected key strength. This is a marked improvement over the standard process of identifying one single preferred method through a quick visual inspection and then either relying on this single method or painstakingly verifying visually that it has performed optimally on each of the several hundred images.

As more segmentation techniques become publicly available, they will be incorporated into CellSampler, ensuring that the pipeline shown in this study continually evolves and maintains quality alongside the field’s latest cutting-edge advances. Future work will also include extending the uber mask functionality from 2D to 3D. This can be implemented in a straightforward manner by either realigning 2D segmentation masks back into 3D volumes or adapting CellSampler to use the existing 3D functionality of methods such as Cellpose and StarDist.

## Data Availability

The datasets presented in this article are not readily available because the IMC data used in this paper, which are not already publicly available, belong to the Cancer Research UK Cambridge Institute. Requests to access the datasets should be directed to dario.bressan@cruk.cam.ac.uk.

## Footnotes

1
https://zarr.readthedocs.io

2
https://parquet.apache.org/

3
https://scikit-image.org/
